# Hepatitis B vaccination using a dissolvable microneedle patch is immunogenic in mice and rhesus macaques

**DOI:** 10.1002/btm2.10098

**Published:** 2018-08-27

**Authors:** Monica B. Perez Cuevas, Maja Kodani, Youkyung Choi, Jessica Joyce, Siobhan M. O'Connor, Saleem Kamili, Mark R. Prausnitz

**Affiliations:** ^1^ School of Chemical and Biomolecular Engineering Georgia Institute of Technology Atlanta GA 30332; ^2^ Division of Viral Hepatitis, National Center for HIV/AIDS, Viral Hepatitis, STD and TB Prevention Centers for Disease Control and Prevention Atlanta GA 30329; ^3^ Wallace Coulter Department of Biomedical Engineering at Georgia Tech and Emory University Georgia Institute of Technology Atlanta GA 30332

**Keywords:** biomechanics, birth dose, hepatitis B surface antigen vaccine, hepatitis B virus, microneedle patch, mouse, rhesus macaque, vaccination

## Abstract

Chronic Hepatitis B virus infection remains a major global public health problem, accounting for about 887,000 deaths in 2015. Perinatal and early childhood infections are strongly associated with developing chronic hepatitis B. Adding a birth dose of the hepatitis B vaccine (HepB BD) to routine childhood vaccination can prevent over 85% of these infections. However, HepB BD coverage remains low in many global regions, with shortages of birth attendants trained to vaccinate and limited HepB BD supply at birth. To address the challenges, we developed coated metal microneedle patches (cMNPs) and dissolvable microneedle patches (dMNPs) that deliver adjuvant‐free hepatitis B vaccine to the skin in a simple‐to‐administer manner. The dMNP contains micron‐scale, solid needles encapsulating vaccine antigen and dissolve in the skin, generating no sharps waste. We delivered HepB BD via cMNP to BALB/c mice and via dMNP to both mice and rhesus macaques. Both cMNP and dMNP were immunogenic, generating hepatitis B surface antibody levels similar to human seroprotection. Biomechanical analysis showed that at high forces the microneedles failed mechanically by yielding but microneedles partially blunted by axial compression were still able to penetrate skin. Overall, this study indicates that with further development, dMNPs could offer a method of vaccination to increase HepB BD access and reduce needle waste in developing countries.

**Abbreviations**
anti‐HBs: antibody to hepatitis B surface antigen
CMC: carboxymethylcellulose
CDC: U.S. Centers for disease control and Prevention
cMNP: coated microneedle patch
dMNP: dissolvable microneedle patch
ELISA: enzyme‐linked immunosorbent assay
HBsAg: hepatitis B surface antigen
HBV: hepatitis B virus
IACUC: Institutional Animal Care and Use Committee
IgG: immunoglobulin G
IM: intramuscular
MNP: microneedle patch
PBS: phosphate‐buffered saline.

## 1 INTRODUCTION

Hepatitis B virus (HBV) infection is a major global health problem. Worldwide, in 2015 an estimated 257 million persons were living with HBV and over 887,000 deaths were attributed to HBV, the majority due to chronic HBV‐associated liver disease (cirrhosis) and liver cancer (hepatocellular carcinoma).^1^, ^2^ Perinatal infection (around birth, as from an HBV‐infected mother) and early childhood infection are major risk factors for the development of chronic HBV infection after exposure.^1^ Therefore, the recent World Health Organization (WHO) goal to eliminate viral hepatitis, including Hepatitis B, as a public health threat by 2030, aims to reduce HBV prevalence among children under 5 years to 0.1% within the goal of reducing new HBV infections by 90%; HBV elimination can only be achieved by eliminating perinatal and early childhood infections.^3^

Successful and widespread implementation of the WHO‐recommended safe and effective immunization against HBV, consisting of delivering a birth dose of the hepatitis B vaccine (HepB BD) followed by timely receipt of the routine childhood HepB vaccine series, is a critical component of the elimination strategy. While global coverage of the routine HepB series reached 84% in 2015, addition of the HepB BD lags far behind at 39%, providing incomplete coverage.^1^ Often the areas with low HepB BD delivery rates are also areas with higher population (including maternal) rates of HBV infection, leaving many infants at risk for early childhood infection with subsequent HBV‐associated cirrhosis and liver cancer. The still frequent occurrence of births outside of larger health facilities that have skilled birth attendants trained to administer HepB BD and unreliable vaccine supply and storage at the site of births contribute substantially to the lower rates of HepB BD delivery in these regions.^4^ This is particularly true in parts of Africa, where only about 60% of births in 2016 were attended by a skilled health professional who would not always be trained in or have access to timely delivery of the hepatitis B BD.^5^, ^6^

Further increasing the demands on training, logistics and cost, current HepB BD and routine HepB delivery require intramuscular (IM) injection, generating the risk for unsafe needle injection (itself a risk factor for HBV infection) and disposal of needle sharps waste. While particularly relevant to limited resource settings, injection safety and disposal of biohazardous waste from injections, especially needles, remain a global concern, even in economically developed regions.

Microneedle patches (MNPs) have been proposed as an alternate mode of vaccination. These patches consist of micron‐scale, solid needles capable of puncturing across the skin's stratum corneum barrier to deliver vaccines into the epidermis and superficial dermis layers of the skin without the need for hypodermic needles.^7^, ^8^, ^9^, ^10^ The microneedles can be made of biocompatible, water‐soluble materials that encapsulate the vaccine and dissolve in the skin, thereby generating no sharps waste.^11^, ^12^ Because they are small, simple‐to‐administer and often thermostable without refrigeration, MNPs could be administered by minimally trained birth attendants, thereby enabling increased coverage of the hepatitis B BD.

The high density of antigen‐presenting cells in the skin make intradermal immunization via MNPs advantageous. For example, prior studies have shown dose sparing, longer‐lived immune responses, greater breadth of immunity, isotype class switching and other immunologic advantages and differences when vaccinating in skin using MNPs as opposed to IM or subcutaneous injection.^13^, ^14^ While microneedles do not typically insert fully into skin due to skin surface deformation, prior studies indicate that insertion is sufficient to access both epidermis and at least superficial dermis, thereby accessing Langerhans cells and dermal dendritic cells, respectively.^15^ In this work, we use the term “intradermal” immunization to refer to stratum corneum puncture accompanied by delivery of vaccine into the epidermis and dermis.

A number of studies have investigated different methods of intradermal hepatitis B vaccine delivery, notably to increase immunogenicity in IM vaccine non‐responders.^16^, ^17^ These studies have incorporated aluminum adjuvants into the vaccine preparations, which have been shown to cause substantial irritation when administered to the skin.^18^, ^19^, ^20^, ^21^ Several studies in animal models have delivered the vaccine intradermally by jet injection, which yielded immune responses comparable to subcutaneous or IM injection.^18^, ^22^ MNPs have also been used in animal studies to administer hepatitis B vaccines using microneedles as a pretreatment to permeabilize skin, followed by topical application of a vaccine‐containing solution,^23^, ^24^, ^25^ by coating metal microneedles with vaccine^26^ and by encapsulating vaccine in dissolving MNPs.^27^, ^28^

A critical aspect of MNP design is the use of materials and microneedle geometry that enable reliable insertion into skin without mechanical damage to the microneedles. Microneedle design also needs to account for skin deformation during microneedle insertion, which can be accomplished by using microneedles of sufficient length to account for deformation and maximal sharpness to minimize that deformation.^12^, ^29^ Insertion of shorter microneedles can be performed at a high velocity that allows skin penetration to occur on a timescale faster than the mechanical relaxation time of the skin.^30^, ^31^, ^32^ Failure of microneedles due to the compressive forces present during insertion into skin has been studied experimentally and modeled.^33^, ^34^, ^35^, ^36^, ^37^, ^38^ These studies show that microneedles can be designed to insert into skin at forces substantially lower than those associated with microneedle fracture.

In this study, we developed MNPs to administer adjuvant‐free hepatitis B surface antigen (HBsAg) vaccine, evaluated their mechanical properties in the context of skin insertion and mechanical failure, and studied their immunogenicity in mice and rhesus macaques.

## 2 MATERIALS AND METHODS

### 2.1 Concentration of hepatitis B vaccine

Concentrated bulk solution of aluminum adjuvant‐free hepatitis B vaccine (HBsAg) was generously provided by the Serum Institute of India (Pune, India). Starting antigen concentration was 2.4 mg/mL. The bulk HBsAg was further concentrated using Vivaspin 20 centrifuge spin filters with a 10 kDa molecular weight cutoff (GE Life Sciences, Pittsburgh, PA) by centrifugation at 700 g for 2 min. HBsAg bulk was concentrated 4‐fold (9.5 mg/mL) and 5.4‐fold (12.9 mg/mL) for the dissolvable and the metal microneedles, respectively (see below). For our initial mouse study, unmodified bulk HBsAg (2.4 mg/mL) was used instead of the concentrated HBsAg solution. HBsAg concentration was measured via the VITROS Immunodiagnostic System (Ortho Clinical Diagnostics, Raritan, NJ).

### 2.2 Microneedle patch fabrication

#### 2.2.1 Coated microneedle patches

In‐plane rows of stainless steel microneedle arrays were coated using an in‐house dip‐coating device to make coated MNPs (cMNPs). The microneedle arrays were fabricated by Tech Etch (Plymouth, MA). Each array consisted of five microneedles measuring 750 µm in length and 170 µm by 50 µm in base cross‐sectional area, and arranged in a linear array with 1.6 mm microneedle tip‐to‐tip spacing. Two linear micro‐positioners allowed for alignment and dipping of individual microneedles into a micropipette tip containing a coating solution. The coating solution consisted of concentrated adjuvant‐free HBsAg (Serum Institute of India) mixed 1:1 with a solution of 2% w/v (weight per volume) carboxymethylcellulose (CMC, Sigma–Aldrich, St. Louis, MO) and 20% w/v trehalose (Sigma–Aldrich). This device design allowed for coating of only the microneedle shaft, thereby avoiding contamination of the array base. Elutions of microneedle coatings in 2.5 mL of 0.2% w/v bovine serum albumin (BSA) in phosphate‐buffered saline (PBS) per array were assayed by VITROS HBsAg test kit to estimate antigen dose delivery.

#### 2.2.2 Dissolvable microneedle patches

Dissolvable microneedle patches (dMNPs) were fabricated as described previously.^39^, ^40^ In this study, the adjuvant‐free HBsAg vaccine was mixed into a casting solution at a 60:40 ratio with casting solution containing 25% w/v trehalose and 2.5% w/v CMC in PBS. For the mouse study, unmodified bulk HBsAg was used instead of the concentrated solution. Molds used to fabricate dMNPs consisted of a 10 × 10 array of conical cavities in the shape of microneedles. 35 µL of the vaccine casting solution was applied to each mold, after which “excess” vaccine solution was scraped off, leaving ∼7 µL of casting solution to dry into the mold. This corresponded to a dose of ∼40 µg HBsAg applied to each mold. The needle portion of the dMNP makes up about 60% of this volume, making for 24 µg of administrable HBsAg.

After drying for 1 hr at room temperature (20–25°C) and 20–50% relative humidity, a second casting solution was applied to form a patch backing. The backing solution consisted of sucrose (Sigma–Aldrich) and poly‐vinyl alcohol (Acros Organics, NJ) dissolved in deionized water at 25°C for 1 hr to a final concentration of 0.53 mg/mL of each solute. Vacuum was applied to the mold at room temperature (20–25°C) for 3 hr. After that, molds were allowed to further dry in a desiccator at room temperature for 2 days. dMNPs prepared for mechanical testing experiments contained PBS instead of vaccine.dMNPs were removed from the mold by applying a polymethylmethacrylate (McMaster‐Carr, Atlanta, GA) disk covered with double‐sided tape (MacTac, Stow, OH) to the back of the mold and slowly pulling the patch away from the mold. Upon removal, dMNPs were stored inside a sealed pouch with desiccant (Drierite, Xenia, OH) at 4°C for up to 24 hr until use. To avoid damaging the microneedles through condensation of atmospheric water, pouches were allowed to return to room temperature prior to patch removal and application. Dissolution of dMNPs in 2.5 mL of 0.2% w/v BSA in PBS per patch were assayed by VITROS HBsAg test kit to estimate dose delivery.

### 2.3 Mechanical characterization

#### 2.3.1 Force‐displacement

dMNPs were kept inside sealed pouches with desiccant until testing. Failure forces of dMNPs under an axial load were measured utilizing a digital force gauge (MARK‐10 Series 5, Copiague, NY). Force‐displacement curves were generated by measuring both force and displacement as the test station's moving crosshead pressed against the dMNP at a rate of 1.0 mm/min. Upon reaching a specific load, the crosshead was immediately lifted. dMNPs were then stored inside their original pouches with desiccant for at least 24 hr at room temperature before subsequent insertion into skin. Height loss of dmNPs was determined by measuring individual microneedles before and after compression using photo editing software (Photoshop, Adobe Systems, San Jose, CA). While data were collected on a continuous basis, we present the data as a series of discreet points on a force‐displacement graph to facilitate averaging among multiple replicate measurements.

A yield point was also determined for each force‐displacement curve, defined as the first detected change in the force‐displacement curve slope, which we consider to be the lowest force at which detectable deformation of the microneedle tip takes place. We call this a yield force rather than a fracture force because microscopic examination of the microneedles indicated a deformation in the tips of the microneedles rather than breaking off of microneedle tips (see “Section 3”). We did not study the mechanical properties of stainless steel cMNPs because previous work has shown the ability of stainless steel cMNPs to reliably insert into skin without deformation or fracture.^41^

#### 2.3.2 Microneedle insertion

Sections of frozen pig skin (Holifield Farms, Covington, GA) were thawed by placing in water (while still inside the bag) and then placed on absorbent pads on top of a rigid lab bench surface to dry under air at room temperature. A Kimwipe (ThermoFisher, Waltham, MA) was placed on top of the skin section to visually determine if the skin surface was dry. dMNPs were inserted into the dried skin within 20 min of removal from the plastic bag if the Kimwipe indicated a dry skin surface. dMNPs previously subjected to specified force loads were applied to the skin held taut by hand while applying an axial force by thumb of approximately 7 N to the dMNP and held for 30 seconds. This method of insertion simulates the intended use of the dMNPs, for example, as used in a recent clinical trial of influenza vaccination.^42^ Twenty minutes later, the dMNP was removed and gentian violet dye (HUMCO, Texarkana, TX) was applied to the skin to stain puncture spots on the stratum corneum. The skin was imaged using an Olympus SZX16 microscope (Tokyo, Japan) to determine insertion efficiency. For this work, we did not study antigen release kinetics of cMNPs and dMNPs. Instead, we relied on previous work from our group showing that 20 min insertion time is enough to achieve complete delivery (i.e., longer insertion time does not further increase delivery) for cMNPs and dMNPs of similar composition.^41^, ^42^

### 2.4 Mouse immunogenicity study

The immunogenicity of adjuvant‐free HBsAg vaccine patches was tested in forty 11‐week‐old female BALB/c mice (Charles River, Wilmington, MA) divided into five groups of eight mice each to receive: (a) IM injection of bulk HBsAg; (b) IM injection of reconstituted dMNP; (c) IM injections of reconstituted cMNP; (d) two dMNPs applied to skin for 10 min per array; (e) two cMNPs applied to skin for 10 min per array. MNPs were reconstituted in saline for injection (Hospira, Lake Forest, IL). The backs of mice receiving MNP vaccination were shaved with electric shears followed by application of a depilatory cream (Nair, Princeton, NJ) one day before vaccination. The mice were anesthetized using isoflurane for the vaccination and blood collection.

All mice received two vaccine doses (by the same method of delivery) separated by 3 weeks. Estimated HBsAg doses administered to the mice were 2.5 µg, 1.1 ± 0.1 µg, 1.9 ± 0.04 µg, 1.2 ± 0.3 µg (2 patches) and 1.1 ± 0.2 µg (2 patches) HBsAg for groups 1–5, respectively. Dosing variability may be related to fabrication variability and/or analysis of concentrations at the limits of the platform measuring range. Because ∼40 µg of HBsAg was applied onto each dMNP mold during fabrication, only ∼1.5% of the applied vaccine was administered to the animals in an active form. Based on measurements of HBsAg in patches before and after application to skin, these losses in vaccine activity mostly occurred while making the dMNPs using a non‐optimized formulation and fabrication method, although ∼30% loss was due to incomplete delivery from the dMNPs into the skin. The goal of this study was to conduct an initial assessment of HBsAg immunogenicity in animals and not to develop an optimized dMNP.

**Figure 1:**
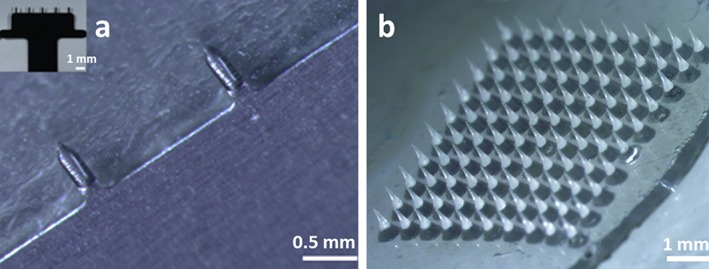
Microneedle patches. (a) Two microneedles from a coated MNP are shown with a coating containing adjuvant‐free HBsAg vaccine localized to the microneedles but not the base substrate. The inset shows a complete coated MNP containing 5 microneedles. (b) A dissolvable MNP composed of a 10 × 10 array of microneedles encapsulating the same HBsAg vaccine

Visual evidence of skin punctures by dMNPs was noticeable (to a trained eye) immediately after administration; no bleeding was observed. Mice were re‐shaved and depilated prior to the second vaccination using MNPs. Blood was collected at weeks 2, 4, 5, and 8 to measure antibody to HBsAg (anti‐HBs) as described in Section 2.6. After week 8, the mice were sacrificed by isoflurane euthanasia. The protocol for these experiments was approved by the Institutional Animal Care and Use Committee (IACUC) at Georgia Tech.

### 2.5 Macaque immunogenicity study

The immunogenicity of HBsAg vaccine administered by dMNPs was tested in rhesus macaques (Covance, Denver, PA). Animals were divided into four groups of four macaques each: (a) IM vaccination with bulk, adjuvant‐free stock HBsAg; (b) IM vaccination with the commercially available, standard aluminum adjuvant monovalent HBsAg vaccine (Serum Institute of India); (c) three dMNPs; (d) six dMNPs. Four animals were used in each group to provide representative replicate values; the study was not powered to detect differences in any particular outcome.

Macaques were anesthetized via ketamine injection during vaccination and collection of blood. For macaques receiving dMNP administration, their backs were shaved using electric shears followed by application of a depilatory cream (Nair, Ewing, NJ). Patches were applied similarly to the ex vivo pig skin experiments by keeping the hairless skin section taut with one hand and pressing by thumb with the other hand for 30 s against the skin. Patches were then left on the skin for 20 min. Blood was collected weekly and analyzed for anti‐HBs titers as described below. The protocol for these experiments was approved by the IACUCs at CDC and Georgia Tech.

All macaques received one dose of vaccine. Estimated HBsAg doses were 10 µg, 10 µg (plus alum), 24 ± 8 µg (3 patches) and 48 ± 14 µg HBsAg (6 patches) per animal for groups I–IV, respectively. Elevated HBsAg dosing by dMNPs was due to unanticipated efficiency in vaccine encapsulation and delivery.

### 2.6 HBV serology

In macaque studies, HBsAg was measured using a modified protocol for quantitative detection of HBsAg in human serum, VITROS HBsAg Reagent Pack and VITROS Immunodiagnostic Products HBsAg Calibrator on the VITROS ECi/ECiQ Immunodiagnostic System (VITROS HBsAg, Ortho Clinical Diagnostics). The assay (reported analytical sensitivity of 0.085 IU/mL^43^) was performed according to the manufacturer's recommendation, and it was modified by addition of a standard curve to quantify HBsAg in MNPs. The standard curve was generated from the aluminum adjuvant‐free HBsAg vaccine stock by serial dilutions in 0.2% w/v BSA in PBS. Furthermore, the quantitative anti‐HBs assay was performed as recommended by the manufacturer using the VITROS Anti‐HBs Reagent Pack and VITROS Immunodiagnostic Products Anti‐HBs Calibrator on the VITROS ECi/ECiQ Immunodiagnostic System (limit of detection of 4.2 mIU/mL). All samples with anti‐HBs >1000 mIU/mL were diluted in Sample Diluent (VITROS anti‐HBs, Ortho Clinical Diagnostics) and re‐run.

In mouse studies, serum samples were analysed with MONOLISA™ Anti‐HBs EIA (Bio‐Rad, Hercules, CA), designed for qualitative and quantitative detection of anti‐HBs in human serum and EDTA or citrated plasma (Bio‐Rad, Hercules, CA). The limit of detection of this assay was 2 mIU/mL.

### 2.7 Statistics

Statistics were performed using Prism software version 7 (GraphPad, La Jolla, CA). *p* values < .05 were considered significant. Mouse experimental data were analyzed each week via one‐way ANOVA with a Tukey post‐test; a two‐way ANOVA with a Tukey post‐test was used to compare macaque experimental data.

## 3 RESULTS

### 3.1 Mechanical characterization of microneedle patches

**Figure 2:**
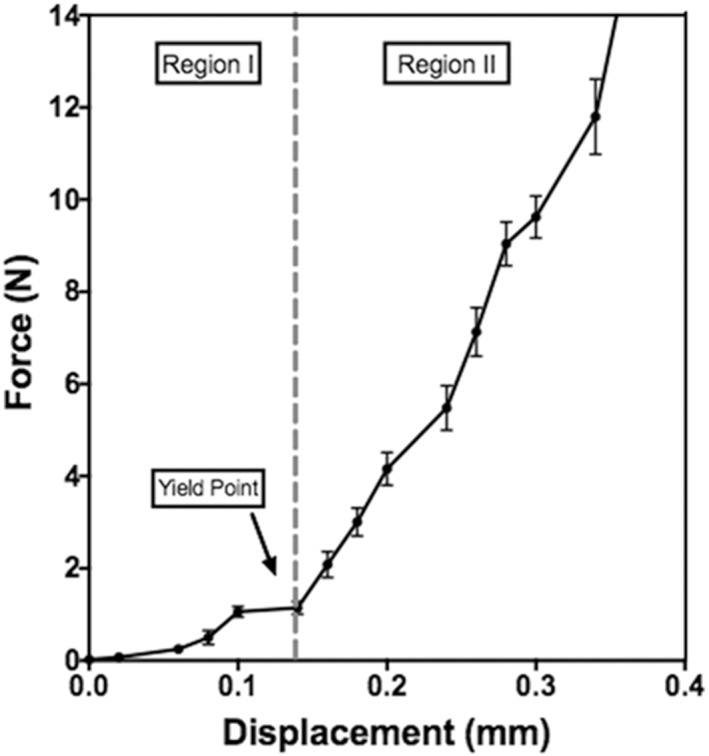
Representative force‐displacement curve for a dissolvable microneedle patch containing a 10 × 10 array of microneedles

While stainless steel microneedles have been shown to have sufficient mechanical strength to penetrate into skin,^44^, ^45^ dissolving polymer microneedles require more careful optimization to assure mechanical strength. We therefore characterized the mechanical properties of the dMNPs used in this study. In response to pressing dMNPs against a rigid aluminum block at a constant rate, force generally increased with increasing applied displacement, which could be divided into two regions (Figure 2). Based on data collected from 11 dMNPs, we found that at small displacement (i.e., less than 0.15 ± 0.05 mm; Region I) there was a progressive increase in force that led to an apparent yield point at 1.1 ± 0.6 N of axial force applied to 100 microneedles (i.e., 11 ± 6 mN per microneedle). Above this yield point, force increased as a much stronger function of applied displacement (Region II).

**Figure 3:**
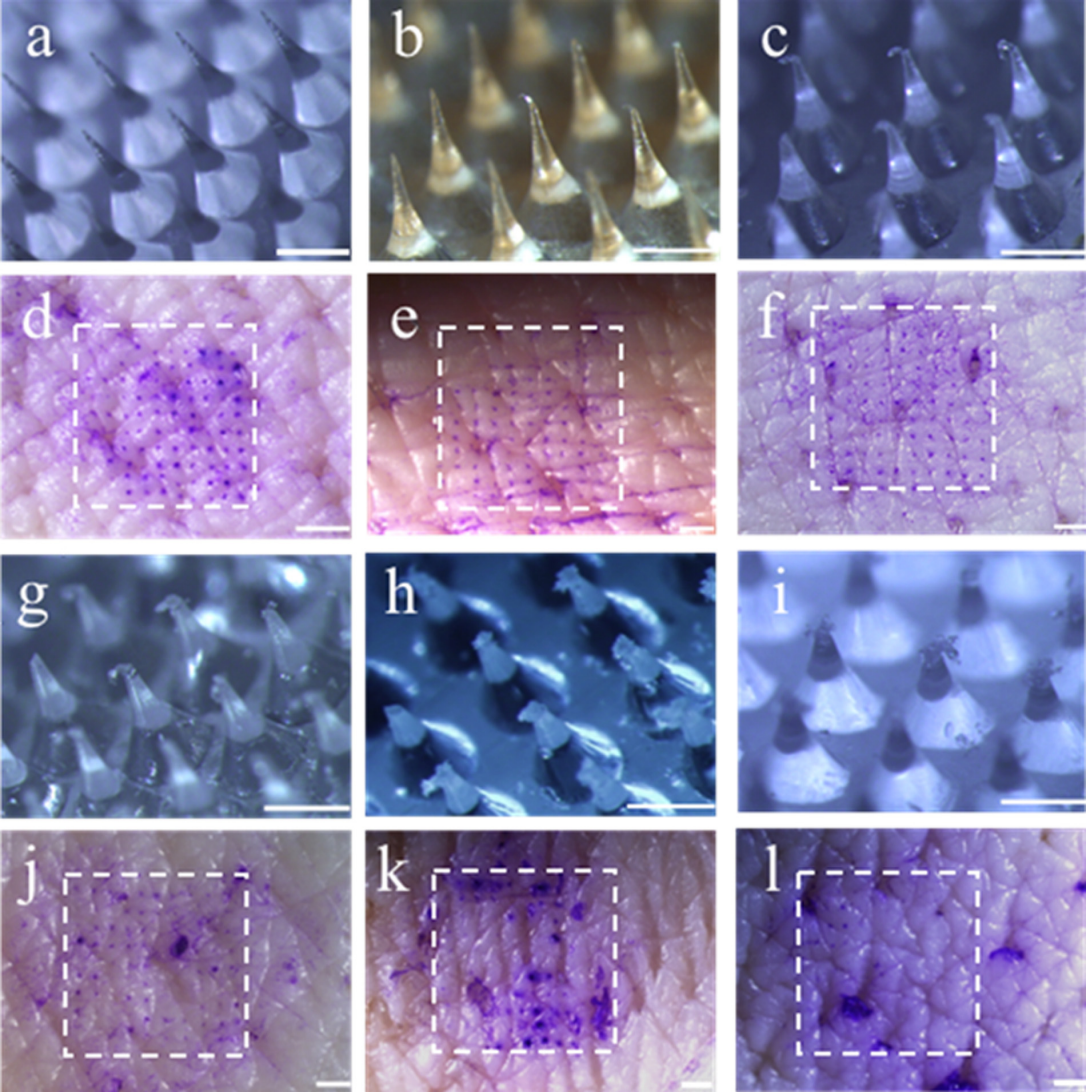
Microneedle deformation and skin insertion after application of axial forces to dissolvable microneedle patches (dMNPs). Representative images of microneedle tip deformation are shown after application of (a) 0 N, (b) 5 N, (c) 15 N, (g) 25 N, (h) 50 N and (i) 75 N to dMNPs containing 100 microneedles. Scale bars are 500 µm. After force application, dMNPs were applied to pig skin ex vivo and sites of microneedle penetration into skin were identified by staining with gentian violet dye. Representative images of skin are shown after application of dMNPs each containing 100 microneedles that had previously been deformed by application of (d) 0 N, (e) 5 N, (f) 15 N, (j) 25 N, (k) 50 N and (l) 75 N. Sites of patch application are identified by a dashed white box. Scale bar: 1 mm

dMNPs were imaged following application of different axial forces. Compared to unused dMNPs (Figure 3a), application of 5 N (above the transition into Region II) revealed initial evidence of microneedle tip deformation (Figure 3b). At higher forces of 15–75 N (Figure 3c,g–i), progressively greater microneedle tip deformation was seen. For the dMNP designs and conditions used, microneedle fracture was not observed.

Comparing the forces associated with changes in measured mechanical properties (Figure 2) with morphological changes observed microscopically (Figure 3), it appears that the mechanics in Region I of the force‐displacement curve correspond to behavior of intact microneedles and that the yield point may correspond to the point of initial microneedle tip deformation (occurring between Figure 3a,b). Region II appears to correspond to progressively increasing microneedle tip deformation (occurring between Figure 3b,c) and, at higher forces microneedle tips appear crushed and the matrix material begins to fan out in a radial direction (Figure 3g–i).

While microneedle tip deformation should not be good for insertion into skin, it may be that some deformation is acceptable. We therefore assessed the ability of microneedles to penetrate into skin after different amounts of tip deformation. Intact microneedles were able to penetrate the skin efficiently, as indicated by an assay that stains sites of microneedle penetration into skin (Figure 3d). Although minor chipping and hooking of the tip occurred after dMNP compression at 5 N and 15 N, subsequent insertion of the dMNPs into skin had similar performance compared to that of an intact patch (Figure 3e,f). After compression at 25 N and 50 N, increased bending and tip deformation was associated with a loss of insertion efficiency, as evidenced by the reduction of penetration holes (Figure 3j,k). Finally, at 75 N, microneedle tips were crushed and no longer able to penetrate skin (Figure 3l).

**Figure 4:**
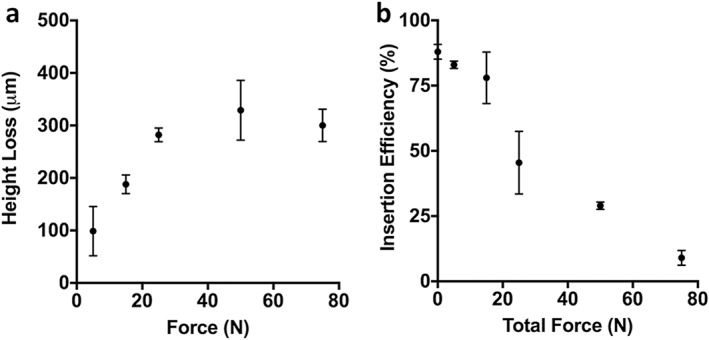
Quantification of microneedle deformation and skin insertion after application of axial forces to dissolvable microneedle patches (dMNP)s. (a) Height loss of microneedles after force application to originally 600 µm tall dMNPs. (b) ex vivo insertion efficiency into pig skin after force application to dMNPs. Insertion efficiency was defined as the percentage of the 100 microneedles on a dMNP that penetrated the skin as determined by counting the number of skin penetrations from images like those shown in Figure 4. (mean ± SD, n = 11)

Quantification of these data showed that microneedle height decreased with increasing force, (one‐way ANOVA, *p* < .0001) (Figure 4a). The increase in microneedle tip diameter that accompanied loss of microneedle height resulted in a blunt tip that progressively inhibited insertion with increasing bluntness (Kruskal‐Wallis rank ANOVA, *p* < .01, Figure 4b), though crushed tips with up to 200 µm height loss (i.e., patches compressed with up to 15 N) still had insertion efficiency >75% (Figure 4b). The acceptable degree of insertion efficiency loss will depend on the application, but the important point of these findings is that minor tip blunting may be tolerated and that major tip blunting only occurred at forces higher than those usually associated with successful microneedle insertion into skin.^11^, ^29^, ^46^

### 3.2 Immunogenicity in mice

**Figure 5:**
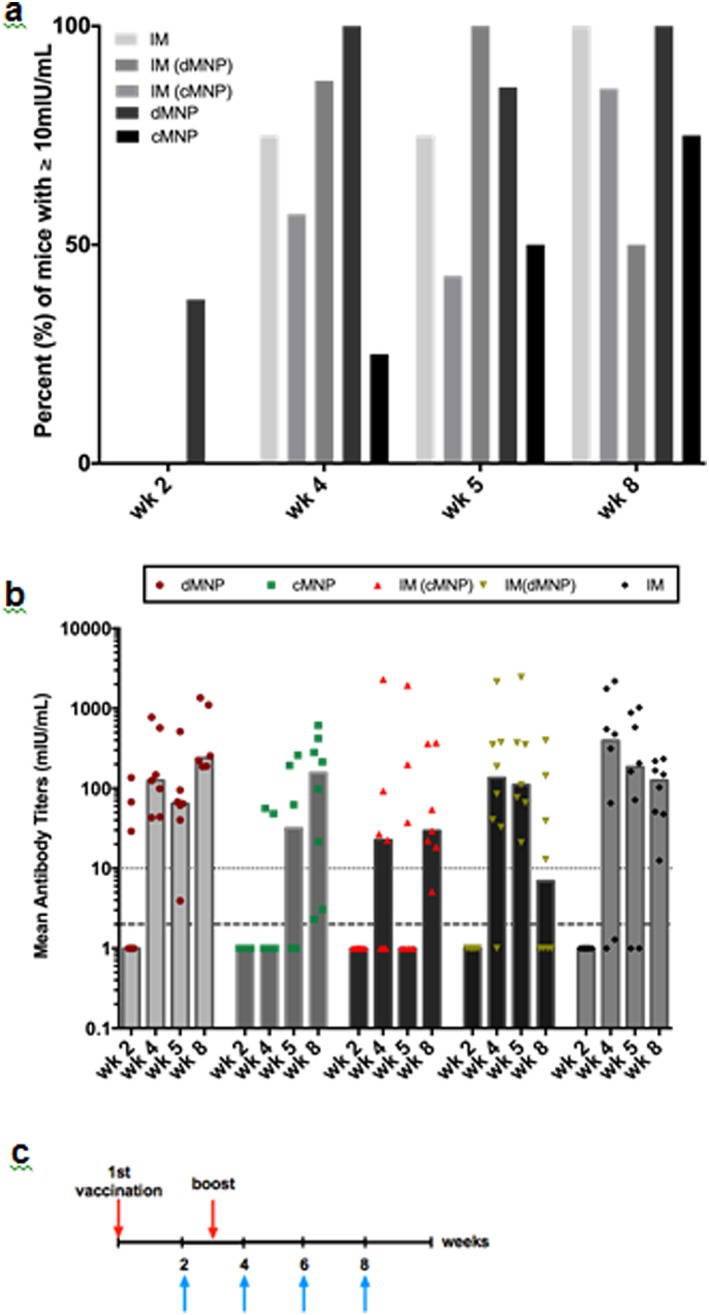
Antibody responses in mice. (a) Percent of BALB/C mice with serum antibody response ≥10 mIU/mL anti‐HBs at 2–8 weeks after vaccination using bulk adjuvant‐free HBsAg vaccine. Vaccine was administered using dissolvable microneedle patches (dMNP) and coated microneedle patches (cMNP), as well as intramuscular (IM) injections of bulk, adjuvant‐free HBsAg vaccine, IM reconstituted dMNPs (IM (dMNP) and IM reconstituted cMNPs (IM (cMNP)). In the latter two groups, dMNPs and cMNPs were dissolved in 270 µL and 300 µL of saline for injection, respectively (i.e., reconstituted) and the eluted vaccine was injected IM. Mice were vaccinated at week 0 and again at week 3. (n = eight mice per group). *(b)* Median anti‐HBs titers. Dotted line indicates the seroprotective level in humans (≥10 mIU/mL anti‐HBs). Dashed line indicates the detection limit of the anti‐HBs assay (≥2 mIU/mL anti‐HBs), that is, all points below that line were below detection limit. (c) Vaccination schedule for all mouse groups. Upper arrows indicated vaccination times. Lower arrows indicate times when blood was sampled

We next administered adjuvant‐free HBsAg using dMNPs and cMNPs to characterize the magnitude of anti‐HBs responses (Figure 5). Two weeks after administration of the first vaccine dose, 38% of mice in the dMNP group had antibody responses that surpassed 10 mIU/mL, which in humans is taken to represent seroprotection against HBV infection.^47^ All mice in the cMNP group or the three IM groups receiving bulk HBsAg, HBsAg reconstituted from cMNP or HBsAg reconstituted from dMNP had no detectable antibody titers (i.e., below the detection limit of 2 mIU/mL). The two reconstituted vaccine patch groups were included to determine if anything associated with the MNP fabrication process affected HBsAg immunogenicity (independent of the MNP route of administration to the skin).

One week after the second vaccination (i.e., 4 weeks into the study), all groups had at least 50% of mice with anti‐HBs levels ≥10 mIU/mL, except the cMNP group that had only 25% mice with anti‐HBs levels >10 mIU/mL (Figure 5). At the end of the study after 8 weeks (i.e., 5 weeks after the second vaccine dose), all groups had at least 50% mice with anti‐HBs levels >10 mIU/mL. Overall, no significant differences between groups was found based on one‐way ANOVA analysis (*p* > .05). Overall, we can conclude that HBsAg vaccine administered by cMNPs and dMNPs was immunogenic in mice.

### 3.3 Immunogenicity in rhesus macaques

Guided by outcomes of vaccination using cMNPs and dMNPs in mice, we next vaccinated rhesus macaques using dMNPs, because rhesus macaques are an animal model more closely related to humans and because dMNPs can have immunological^11^ and logistical^48^ advantages over cMNPs. The rhesus macaques were vaccinated with a standard aluminum adjuvanted (HBsAg) vaccine or non‐adjuvanted bulk HBsAg via IM injection or non‐adjuvanted HBsAg by dMNP at two different doses. Baseline blood samples at weeks 1 and 2 before vaccination were negative for anti‐HBs.

**Figure 6:**
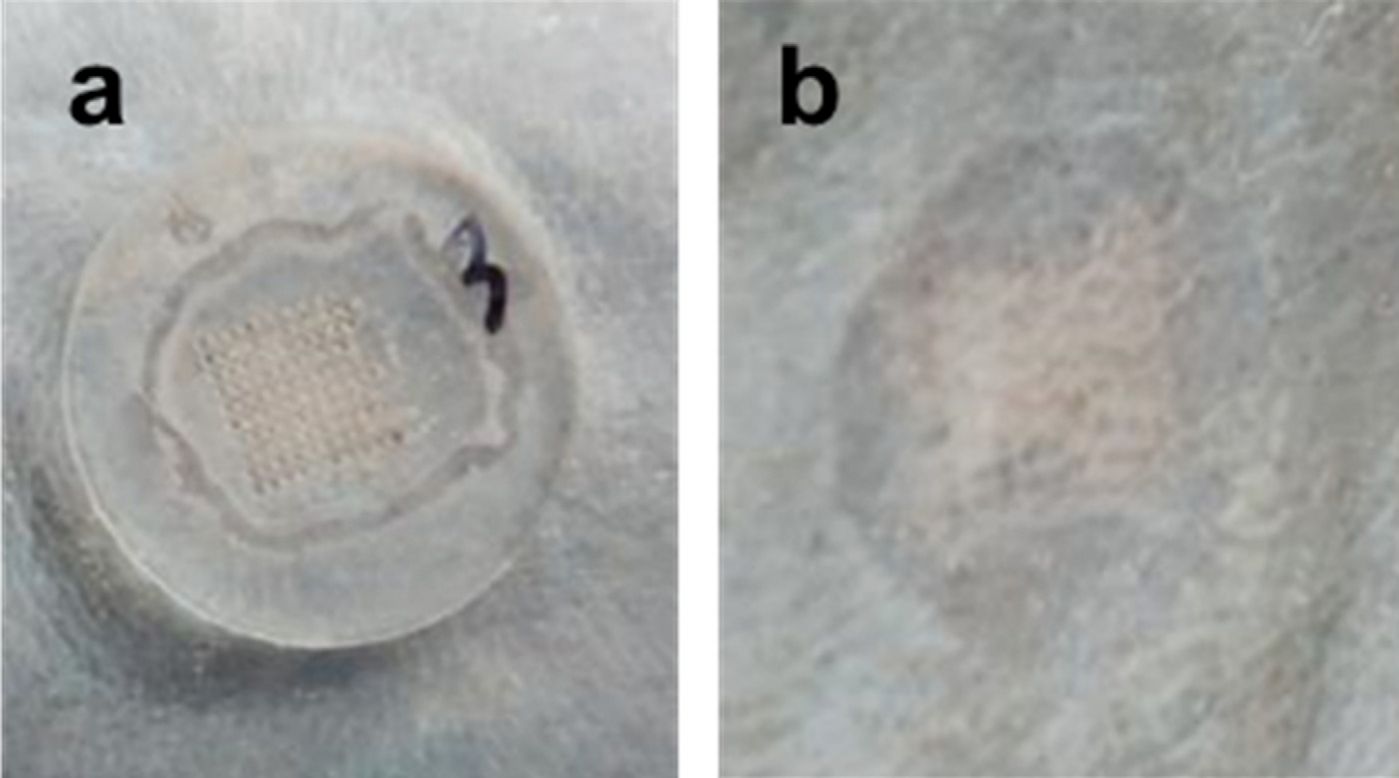
Representative image of dissolvable microneedle patch (dMNP) application to the skin of a rhesus macaque. dMNPs were applied to shaved back skin and left on for 20 min. Images show skin with (a) dMNP in place and (b) immediately after dMNP removal

Immediately after removing the dMNP, there was faint evidence of the site where the microneedles had penetrated the skin (square shape in Figure 6b) and where the adhesive had contacted the skin (round shape in Figure 6b) during patch application. No erythema or other signs of irritation were observed. In ∼10% of the patches, a speck of blood was seen on the residual dMNP post‐administration. No adverse health effects were noted by the veterinary staff.

**Figure 7:**
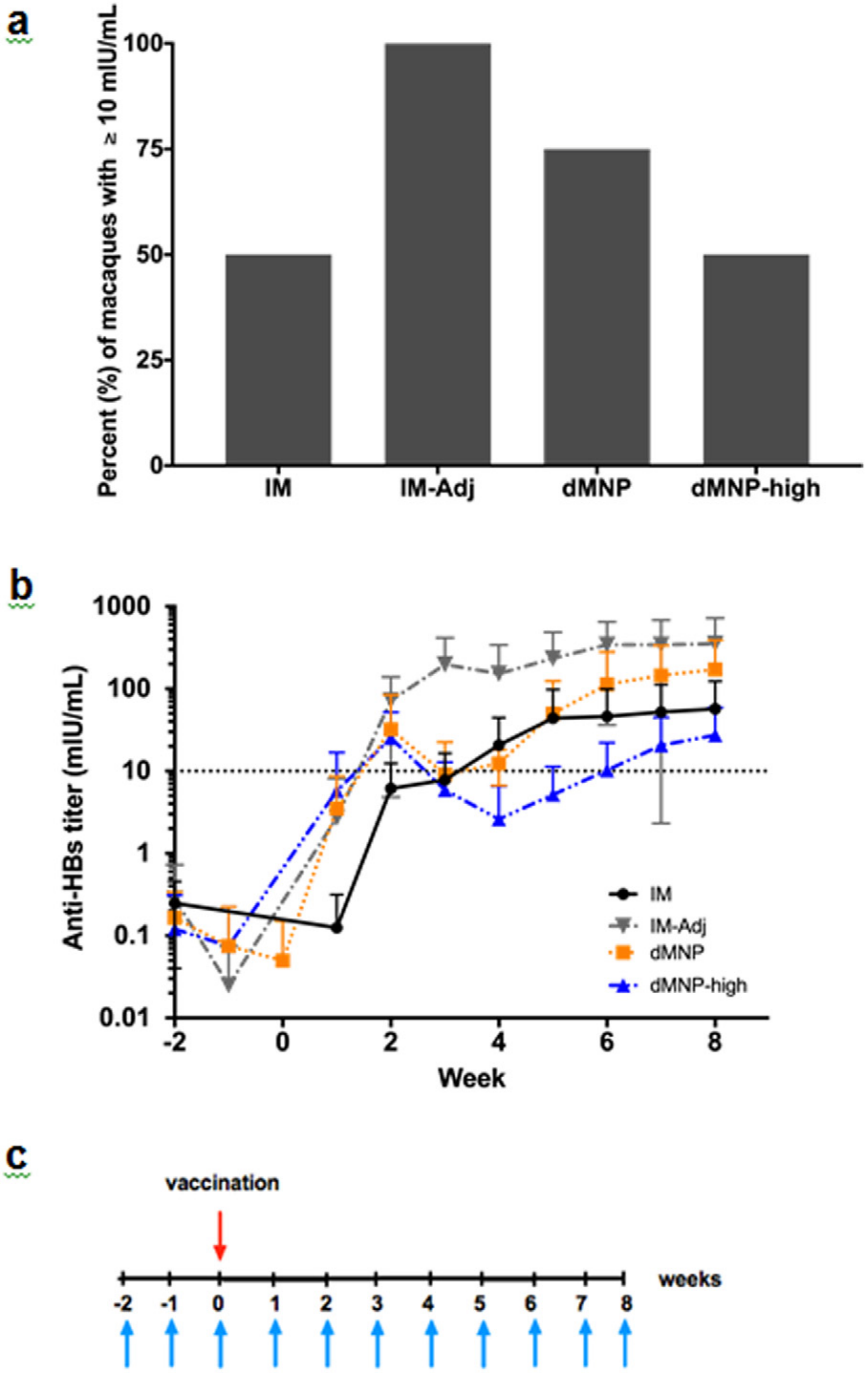
Antibody responses in rhesus macaques. (a) Percent of rhesus macaques with serum antibody response ≥10 mIU/mL anti‐HBs seven weeks after vaccination and (b) mean anti‐HBs titers after vaccination using bulk adjuvant‐free HBsAg vaccine administered IM (IM), commercial HBsAg vaccine‐containing adjuvant administered IM (IM‐Adj) and dMNPs containing a low dose (dMNP) and a high dose (dMNP‐high) of adjuvant‐free HBsAg vaccine. The dotted line indicates the seroprotective level in humans (≥10 mIU/mL anti‐HBs) (mean ± SD, n = 4). (c) Vaccination schedule for all macaque groups. Upper arrow indicates vaccination time. Lower arrows indicate times when blood was sampled

Seven weeks after vaccination, at least half of the macaques (i.e., at least 2 out of 4 macaques) in all groups had anti‐HBs levels ≥10 mIU/mL (considered seroprotective in humans) 100% (4 macaques) in the adjuvanted IM group (IM‐Adj), 75% (3 macaques) in the lower dose dMNP group and 50% (2 macaques) in the adjuvant‐free IM and higher dose dMNP groups (Figure 7a). The humoral immune response was further characterized by examining the anti‐HBs titer kinetics after vaccination (Figure 7b). Antibody responses were not significantly different during the initial immune response up to 4 weeks after vaccination (two‐way ANOVA, *p* > .05). At later times, the adjuvanted IM vaccination group achieved significantly higher anti‐HBs titers (two‐way ANOVA, *p* < .05), but the other three groups had titers that were not significantly different from each other (two‐way ANOVA, *p* > .05) (Figure 7b). By week seven, the anti‐HBs titers for the adjuvanted IM group and the dMNP group were not significantly different (two‐way ANOVA *p* > .05). Overall, these data indicate the dMNP vaccination is similarly immunogenic as IM vaccination without adjuvant, but absolute anti‐HBs levels are less than those detected following IM delivery of adjuvanted vaccine.

## 4 DISCUSSION

### 4.1 Mechanical characterization of microneedles

The dMNPs used in this study exhibited deformation at an apparent yield point of 11 ± 6 mN per microneedle. In this work, we consider yield point to be the lowest force at which noticeable deformation of the microneedle tip takes place as shown be a slope change on the force‐displacement curve. Compared to failure forces measured in prior studies of microneedles, this yield point is comparable to some and significantly lower than others, although direct comparison is difficult because microneedle mechanical strength depends on microneedle geometry, composition and other factors.^30^, ^33^, ^34^, ^36^, ^39^, ^49^ From a practical standpoint, however, microneedles only need to be strong enough to perform their function of penetrating skin, which the microneedles in this study were able to do.

This study addressed, for the first time, the relationship between microneedle tip deformation and insertion into skin. There has been a general expectation that microneedle failure force (i.e., leading to tip deformation) must be significantly larger than the microneedle insertion force for successful skin penetration. While our data largely support that expectation, we found that some level of tip deformation may be acceptable. For the dMNPs used in this study, it was possible to insert microneedles into porcine skin ex vivo after dMNPs experienced forces significantly higher than the observed failure force for failure, which means that this failure force is not the maximum force a microneedle can tolerate before being made unable to insert into skin.

The force required to insert a microneedle into skin depends on microneedle tip sharpness, where smaller forces are needed for sharper tips.^29^ If the yield force of a microneedle is less than the force needed to puncture skin, then the microneedle tip can be blunted while being pressed against the skin but before it inserts into the skin. If this happens, then the blunt‐tipped microneedle requires a still greater force to insert into skin. In this event the microneedle may yield even more as the greater force is applied, thereby increasing the skin insertion force further. This could produce a viscous feedback loop, where increased force (above the microneedle yield force) would cause increased tip deformation, which would increase the microneedle insertion force (due to a less sharp tip), leading to increased tip deformation, etc. In this way, understanding forces associated with microneedle tip deformation and microneedle insertion into skin, which were studied here as separate sequential events, might actually be part of an integrated processes during MNP application to skin.

### 4.2 Immune response to adjuvant‐free HBsAg vaccine delivery using microneedle patches

The main goal of this study was to assess the immunogenicity of dMNP containing HBsAg vaccine, specifically in the absence of aluminum adjuvant, present in all commercially available hepatitis B vaccine preparations. We showed in two animal models–mice and rhesus macaques–that dMNP delivery of this preparation can elicit anti‐HBs responses that exceeded a threshold of ≥10 mIU/mL, levels considered seroprotective in humans.^47^ More specifically, anti‐HBs response following dMNP delivery of adjuvant‐free, bulk vaccine was similar to IM delivery of the adjuvant‐free bulk vaccine in both mice and rhesus macaques. IM delivery of the standard aluminum adjuvanted vaccine produced higher antibody titers. These studies therefore show that dMNPs delivering adjuvant‐free HBsAg vaccine can induce humoral immune responses comparable to IM vaccination, which provides a proof‐of‐principle that dMNPs may be further developed for HBsAg vaccination. Additional studies are needed.

In the dMNP‐high group in the rhesus macaque study, two of the four macaques were non‐responsive to vaccination and the other two animals had anti‐HBs titers ≥10 mIU/mL. It remains unclear why this occurred, as the dMNP‐high patches were shown to be antigenic by the VITROS immunoassay. These inconsistent data may be related to dMNP‐high patch function and/or variability within the small sample size of outbred animals used in this study.

In this study, we evaluated immune responses in adult animals, but were motivated to carry out this study to evaluate possible vaccination using dMNPs in infants, including immediately after birth. Because infants have weaker immune systems than adults,^50^ additional studies will be needed to assess HBsAg vaccination by dMNP in infants, as was done in a recent study of measles and rubella vaccination by dMNP in infant rhesus macaques.^40^

## 5 CONCLUSION

There is a significant need for increased hepatitis B vaccination, especially in infants needing HepB BD in order to prevent perinatal HBV transmission. Hepatitis B vaccine administration using a dMNP could facilitate vaccination, especially in developing countries where trained healthcare personnel are in limited supply and many births are performed without the assistance of a birth attendant trained in IM delivery of the vaccine. dMNPs offer simple‐to‐administer vaccination that generates no sharps waste, which should facilitate hepatitis B vaccination with minimally trained personnel.

In this study, we developed dMNPs that administer the adjuvant‐free bulk stock of a licensed monovalent HBsAg vaccine used for HepB BD immunization in newborns and found that they generated robust anti‐HBs responses in most BALB/c mouse and rhesus macaque animal models in this study. Biomechanical analysis of the dMNPs showed that the microneedles yielded under axial compression. The resulting microneedle tip deformation impeded insertion into skin, but many microneedles could still be inserted into skin even after significant tip deformation. Overall, we conclude that with further development dMNPs may offer a simple‐to‐administer method for hepatitis B vaccination that could be used to give birth doses in the absence of trained healthcare professionals.

## DISCLOSURES

Mark Prausnitz is an inventor of patents that have been or may be licensed to companies developing microneedle‐based products, is a paid advisor to companies developing microneedle‐based products and is a founder/shareholder of companies developing microneedle‐based products, including Micron Biomedical. These potential conflicts of interest have been disclosed and are being managed by Georgia Tech and/or Emory University. The findings and conclusions in this report are those of the author(s) and do not necessarily represent the official position of the U.S. Centers for Disease Control and Prevention.

## References

[1] World Health Organization . Global hepatitis report 2017 http://apps.who.int/iris/bitstream/10665/255016/1/9789241565455-eng.pdf?ua=1. 2017.

[2] World Health Organization . Global health estimates 2015: deaths by cause, age, sex, by country and by region, 2000–2015. http://www.who.int/healthinfo/global_burden_disease/estimates/en/index1.html. Published 2016. Accessed September 27, 2017.

[3] World Health Organization . Global Health Sector Strategies for HIV, viral hepatitis and STIs, 2016–2021. http://www.who.int/hiv/strategy2016-2021/en/. Accessed April 18, 2016.

[4] UNICEF . UNICEF data: monitoring the situation of women and children. Delivery care 2015–2016. https://data.unicef.org/topic/maternal-health/delivery-care/. Published 2017. Accessed September 27, 2017.

[5] World Health Organization . Global health observatory (GHO) data. Skilled attendants at birth. http://www.who.int/gho/maternal_health/skilled_care/skilled_birth_attendance_text/en/. Accessed January 1, 2018.

[6] World Health Organization . Global strategy for women's, children's and adolescents’ health (2016–2030). Proportion of births attended by skilled health personnel (S>D>G>3.12). http://apps.who.int/gho/data/node.gswcah. Accessed January 4, 2018.

[7] Arya J , Prausnitz MR. Microneedle patches for vaccination in developing countries. J Control Release. 2016;240:135–141. 10.1016/j.jconrel.2015.11.019. 26603347 PMC4871790

[8] Marshall S , Sahm LJ , Moore AC. The success of microneedle‐mediated vaccine delivery into skin. Hum Vaccines Immunother. 2016;12(11):2975–2983. 10.1080/21645515.2016.1171440. PMC5137519 27050528

[9] Van Der Maaden K , Jiskoot W , Bouwstra J. Microneedle technologies for (trans)dermal drug and vaccine delivery. J Control Release. 2012;161(2):645–655. 10.1016/j.jconrel.2012.01.042. 22342643

[10] Quinn HL , Kearney M‐C , Courtenay AJ , McCrudden MT , Donnelly RF. The role of microneedles for drug and vaccine delivery. Expert Opin Drug Deliv. 2014;11(11):1769–1780. 25020088 10.1517/17425247.2014.938635

[11] Sullivan SP , Koutsonanos DG , Del Pilar Martin M , et al. Dissolving polymer microneedle patches for influenza vaccination. Nat Med. 2010;16(8):915–920. 10.1038/nm.2182. 20639891 PMC2917494

[12] Arya J , Henry S , Kalluri H , McAllister DV , Pewin WP , Prausnitz MR. Tolerability, usability and acceptability of dissolving microneedle patch administration in human subjects. Biomaterials 2017;128:1–7. 10.1016/j.biomaterials.2017.02.040. 28285193 PMC5382793

[13] Engelke L , Winter G , Hook S , Engert J. Recent insights into cutaneous immunization: how to vaccinate via the skin. Vaccine 2015;33(37):4663–4674. 10.1016/j.vaccine.2015.05.012. 26006087

[14] Gill HS , Kang S‐M , Quan F‐S , Compans RW. Cutaneous immunization: an evolving paradigm in influenza vaccines. Expert Opin Drug Deliv. 2014;11(4):615–627. 24521050 10.1517/17425247.2014.885947 PMC4009492

[15] Prausnitz MR. Engineering microneedle patches for vaccination and drug delivery to skin. Annu Rev Chem Biomol Eng. 2017;8(1):177–200. http://www.annualreviews.org/doi/10.1146/annurev-chembioeng-060816-101514. 28375775 10.1146/annurev-chembioeng-060816-101514

[16] Filippelli M , Lionetti E , Gennaro A , et al. Hepatitis B vaccine by intradermal route in non responder patients: an update. World J Gastroenterol. 2014;20:10383–10394. 25132754 10.3748/wjg.v20.i30.10383 PMC4130845

[17] Rivey MP , Peterson J. Intradermal hepatitis B vaccination. 1991;25. 10.1177/106002809102500612 1831579

[18] Hogan NC , Anahtar MN , Taberner AJ , Hunter IW. Delivery of immunoreactive antigen using a controllable needle‐free jet injector. J Control Release. 2017;258:73–80. 10.1016/j.jconrel.2017.05.003. 28479095

[19] Sirinavin S , Muchacheap T , Khupulsup K , Inthraphuwasak W , Petchclai B. Intradermal hepatitis B virus immunization: immunogenicity and reactogenicity. Southeast Asian J Trop Med Public Heal 1991;22:577–580. http://www.ncbi.nlm.nih.gov/entrez/query.fcgi?cmd=Retrieve&db=PubMed&dopt=Citation&list_uids=1820647. 1820647

[20] Wang J , Li B , Wu MX. Effective and lesion‐free cutaneous influenza vaccination. Proc Natl Acad Sci USA. 2015;112(16):5005–5010. http://www.ncbi.nlm.nih.gov/pubmed/25848020%5Cnhttp://www.pnas.org/lookup/doi/10.1073/pnas.1500408112. 25848020 10.1073/pnas.1500408112 PMC4413338

[21] Hickling JK , Jones KR , Friede M , Zehrung D , Chen D , Kristensenc D. Intradermal delivery of vaccines: potential benefits and current challenges. Bull World Health Org. 2011;89:221–226. 21379418 10.2471/BLT.10.079426 PMC3044245

[22] Ploemen IHJ , Hirschberg HJHB , Kraan H , et al. Minipigs as an animal model for dermal vaccine delivery. Comp Med. 2014;64(1):50–54. 24512961 PMC3929219

[23] Hirschberg H , Van Kuijk S , Loch J , et al. A combined approach of vesicle formulations and microneedle arrays for transcutaneous immunization against hepatitis B virus. Eur J Pharm Sci. 2012;46(1–2):1–7. 10.1016/j.ejps.2012.01.013. 22330147

[24] Guo L , Qiu Y , Chen J , Zhang S , Xu B , Gao Y. Effective transcutaneous immunization against hepatitis B virus by a combined approach of hydrogel patch formulation and microneedle arrays. Biomed Microdevices. 2013;15(6):1077–1085. 23893014 10.1007/s10544-013-9799-z

[25] Mikszta JA , Alarcon JB , Brittingham JM , Sutter DE , Pettis RJ , Harvey NG. Improved genetic immunization via micromechanical disruption of skin‐barrier function and targeted epidermal delivery. Nat Med. 2002;8(4):415–419. 11927950 10.1038/nm0402-415

[26] Andrianov AK , DeCollibus DP , Gillis HA , et al. Poly[di(carboxylatophenoxy)phosphazene] is a potent adjuvant for intradermal immunization. PNAS. 2009;106(45):18936–18941. http://www.pubmedcentral.nih.gov/articlerender.fcgi?artid=2770009&tool=pmcentrez&rendertype=abstract. 19864632 10.1073/pnas.0908842106 PMC2770009

[27] Qiu Y , Guo L , Mao P , Gao Y. Dissolving microneedle arrays for intradermal immunization of hepatitis B virus DNA vaccine. Procedia Vaccinol. 2015;9:24–30. http://linkinghub.elsevier.com/retrieve/pii/S1877282X15000053.

[28] Poirier D , Renaud F , Dewar V , et al. Hepatitis B surface antigen incorporated in dissolvable microneedle array patch is antigenic and thermostable. Biomaterials 2017;145:256–265. http://linkinghub.elsevier.com/retrieve/pii/S0142961217305574. 28915391 10.1016/j.biomaterials.2017.08.038

[29] Davis SP , Landis BJ , Adams ZH , Allen MG , Prausnitz MR. Insertion of microneedles into skin: measurement and prediction of insertion force and needle fracture force. J Biomech. 2004;37(8):1155–1163. 15212920 10.1016/j.jbiomech.2003.12.010

[30] van der Maaden K , Sekerdag E , Jiskoot W , Bouwstra J. Impact‐insertion applicator improves reliability of skin penetration by solid microneedle arrays. AAPS J. 2014;16(4):681–684. http://link.springer.com/10.1208/s12248-014-9606-7. 24760438 10.1208/s12248-014-9606-7 PMC4070271

[31] Lahiji SF , Dangol M , Jung H. A patchless dissolving microneedle delivery system enabling rapid and efficient transdermal drug delivery. Sci Rep. 2015;5(1):1–7. 10.1038/srep07914 PMC4300505 25604728

[32] Verbaan FJ , Bal SM , Berg DJVD , Dijksman JA , Hecke MV. Improved piercing of microneedle arrays in dermatomed human skin by an impact insertion method. 2008;128:80–88. 10.1016/j.jconrel.2008.02.009 18394741

[33] Park J‐H , Prausnitz MR. Analysis of mechanical failure of polymer microneedles by axial force. J Korean Phys Soc. 2010;56(4):1223 21218133 10.3938/jkps.56.1223 PMC3016089

[34] Raphael AP , Crichton ML , Falconer RJ , et al. Formulations for microprojection/microneedle vaccine delivery: structure, strength and release profiles. J Control Release. 2016;225:40–52. 10.1016/j.jconrel.2016.01.027. 26795684

[35] Park JH , Allen MG , Prausnitz MR. Biodegradable polymer microneedles: fabrication, mechanics and transdermal drug delivery. J Control Release. 2005;104(1):51–66. 15866334 10.1016/j.jconrel.2005.02.002

[36] Crichton ML , Archer‐Jones C , Meliga S , et al. Characterising the material properties at the interface between skin and a skin vaccination microprojection device. Acta Biomater. 2016;36:186–194. 10.1016/j.actbio.2016.02.039. 26956913

[37] Demir YK , Akan Z , Kerimoglu O. Characterization of polymeric microneedle arrays for transdermal drug delivery. PLoS One. 2013;8:1–9. 10.1371/journal.pone.0077289 PMC3806750 24194879

[38] Ma G , Wu C. Microneedle, bio‐microneedle and bio‐inspired microneedle: a review. J Control Release. 2017;251:11–23. 10.1016/j.jconrel.2017.02.011. 28215667

[39] Lee JW , Park JH , Prausnitz MR. Dissolving microneedles for transdermal drug delivery. Biomaterials 2008;29(13):2113–2124. 18261792 10.1016/j.biomaterials.2007.12.048 PMC2293299

[40] Joyce J, Carroll TD, Collins ML, Chen MH, Fritts L, Dutra JC, et al. A microneedle patch vaccine for measles and rubella is immunogenic in infant rhesus macaques. *J Infect Dis* 2018;218:124–132. 10.1093/infdis/jiy139. PMC5989599 29701813

[41] Gill HS , Prausnitz MR. Coated microneedles for transdermal delivery. J Control Release. 2007;117(2):227–237. 17169459 10.1016/j.jconrel.2006.10.017 PMC1853346

[42] Rouphael NG , Paine M , Mosley R , et al. The safety, immunogenicity, and acceptability of inactivated influenza vaccine delivered by microneedle patch (TIV‐MNP 2015): a randomised, partly blinded, placebo‐controlled, phase 1 trial. Lancet 2017;390(10095):649–658. 28666680 10.1016/S0140-6736(17)30575-5 PMC5578828

[43] Chiron Corp . VITROS Immunodiagnostic products HBsAg instructions for use. 1–18.

[44] Vinayakumar KB , Kulkarni PG , Nayak MM , et al. A hollow stainless steel microneedle array to deliver insulin to a diabetic rat. J Micromech Microeng. 2016;26(6):065013.

[45] Chandrasekaran S , Frazier AB. Characterization of surface micromachined hollow metallic microneedles. J Microelectromech Syst. 2003;12(3):289–366.

[46] Kochhar JS , Quek TC , Soon WJ , Choi J , Zou S , Kang L. Effect of microneedle geometry and supporting substrate on microneedle array penetration into skin. J Pharm Sci. 2013;102(11):4100–4108. 24027112 10.1002/jps.23724

[47] Schillie SF , Murphy TV. Seroprotection after recombinant hepatitis B vaccination among newborn infants: a review. Vaccine 2013;31(21):2506–2516. 10.1016/j.vaccine.2012.12.012. 23257713

[48] Kim YC , Park JH , Prausnitz MR. Microneedles for drug and vaccine delivery. Adv Drug Deliv Rev. 2012;64(14):1547–1568. 10.1016/j.addr.2012.04.005. 22575858 PMC3419303

[49] Sullivan SP , Murthy N , Prausnitz MR. Minimally invasive protein delivery with rapidly dissolving polymer microneedles. Adv Mater. 2008;20(5):933–938. 23239904 10.1002/adma.200701205 PMC3519393

[50] Sanchez‐Schmitz G , Levy O. Development of newborn and infant vaccines. Sci Transl Med. 2011;3(90):90ps27–90ps29. 10.1126/scitranslmed.3001880 PMC4108897 21734174

